# Supplementary material for: Varying isoleucine level to determine effects on performance, egg quality, serum biochemistry, and ileal protein digestibility in diets of young laying hens
Source: PLoS One. 2022 Jan 21;17(1):e0261159. doi: 10.1371/journal.pone.0261159 (PMC8782478; doi:10.1371/journal.pone.0261159)
Supplement: Attachment — Submitted filename: Isoluecine author comments and replies.docx [file pone.0261159.s002.docx]

Varying digestible isoleucine level to determine effects on performance, egg quality, serum biochemistry, and ileal protein digestibility in diets of young laying hens

Response to Reviewers

**Authors wish to thank Reviewers for very helpful comments to improve the reporting of our research.** Author comments in reply to reviewers are **in bold.**

5. Review Comments to the Author

Please use the space provided to explain your answers to the questions above. You may also include additional comments for the author, including concerns about dual publication, research ethics, or publication ethics. (Please upload your review as an attachment if it exceeds 20,000 characters)

Reviewer #1: Reviewer’s comments on Manuscript no PONE-D-20-35024.

The topic is very interesting and does allow knowledge transfer in the field of avian nutrition. However, the manuscript lacks certain details, which must be added. Major revision is required before it can be accepted for publication. The comments below will allow the authors to improve their manuscript:


Abstract:
Line 1 & 2: In control diet, 0.10 isoleucine was added to achieve 0.66% isoleucine levels. Mention the supplemental levels of isoleucine for all diets to achieve 0.66 to 0.84% isoleucine in complete feed.

To ascertain an appropriate level of isoleucine for LSL-LITE layers (23- to 30-week-old), diets containing total isoleucine concentrations (levels) of 0.66 (Control), 0.69, 0.72, 0.75, 0.78, 0.81, and 0.84% were fed as 7 treatments (2730 kcal/kg metabolizable energy) x 7 replicates x 10 birds per replicate.

**In Material and Methods, page 15, lines 6-8.**

**They were fed the Control (basal diet + 0.66% L - isoleucine as calculated, Table 5) and diets containing calculated total percentages of L-isoleucine at 0.69, 0.72, 0.75, 0.78, 0.81, and 0.84%.**

Line 3 & 4: The authors must mention the control diet's crude protein value along with the ME values.

**Materials and Methods, Table 5**

| **ME (Kcal / Kg)** | **2728.00** |
| --- | --- |
| **ME (MJ / Kg)** | **11.41** |
| **Crude Protein** | **16.77** |

Line 6 &7: What is meant by weight change? Do you mean weight gain? Rephrase the term to clarify.

**Weight change = weight gain in Table 1**

Line 12-14: Authors need to do a regression analysis to confirm the optimum levels of isoleucine for commercially important parameters. There may be different optimum levels for different parameters.

**Our goal was not to provide optimum levels for each parameter. The literature is replete with this information.**

**Page 5, lines 1- 4.**

**"In the present work, we explored a range of levels (0.66% to 0.84%) for total isoleucine ( calculated as bound plus synthetic) in the diet to understand how various performance and egg quality parameters** **for** 23- to -30 week-old Lohmann **LSL LITE layers were affected generally and to ascertain a specific level of isoleucine for further study with other BCAA's.''**

**Conclusion**

Data from this study is useful because researchers and producers can conduct regression analyses to determine the optimum level of isoleucine for various parameters of their choice. For the purpose of our work, it was important to determine a specific level of isoleucine for an important parameter to compare to other BCAA's. In the poultry industry, FCR is most often selected as an important performance variable in determining the most appropriate diet (Best, 2011; Clark et al. 2019). **In our work, isoleucine levels at 0.72% and 0.84% produced low FCR of 1.45 and 1.44, respectively, but shared significance of FCR (1.49) with 0.78%. Considering cost of synthetic isoleucine as a dietary ingredient along with the low FCR, 0.72% was chosen for further study.**

The introduction is, not clear and needs to be elaborated and rewritten to justify higher isoleucine levels? What has changed, which demands higher levels?

**Please see new Introduction from pages 2 - 5.**

Line 7-8: “Researchers have proposed optimum isoleucine levels to be from 550 to 660mg/hen /day”: But it is not clear how much of the supplemental isoleucine is needed in the diet to fulfil this requirement? This recommendation is for which breeds and what ages??

**Please see new Introduction from pages 2 - 5.**

Line 8-9: Sentence needs revision. What do authors mean by genetic variation and age groups?

**Please see new Introduction from pages 2 - 5.** Genetic variation refers to breeds.

Table 1: was it for 20 to 30 weeks? Or **23 to 30 weeks**?

Table 1: Units for Egg production and Feed intake is missing?

Table1: Change in bird weight? Not clear? I think authors means initial at minus final wt. If that is the case, then it should be mentioned as weight gain.
Table 1: Egg mass data is missing? Must add.

**Please see corrected Table 1**. I**soleucine level and performance^@^ of layers for 23-30 weeks.**

Page 6: line 2: Bodyweight change was significant for the week? Did it increase or decrease with age

**See p 6, lines 17 and 18.** I**soleucine level and performance^@^ of layers for 23-30 weeks.**

Page 6: Line 3: numerical differences mean nothing? The level of egg production was statistically lower for all treatments fed 0.75 to 0.84% supplemental isoleucine? Revise sentence to clarify what authors are trying to highlight?
Line 4: Highest numerical FCR? This is not true. Numerical differences should not be highlighted. Revise the sentence.

**Page 6, lines 21 to 23 and page 7, lines 1-4**

**"FCR linearly decreased significantly with level; the response was also cubic. Isoleucine at 0.72% and 0.84% significantly reduced FCR compared to all other levels except 0.78%. Da Rocha et al. (2013) reported a quadratic effect of isoleucine level on FCR.** Clark et al. (2019) compared feeding behavior of slected **ISA Brown layers at 25 - 30 weeks of age. Hens with FCR < 1.8 ± 0.02 (high feed efficiency) consumed less feed (as in our work) and preferred a diet with greater ash content and lower gross energy when compared to hens having high FCR > 2.1 ± 0.02 (low feed efficiency)."**

Table 2: Line 5-12: Needs revision: The authors mention that levels and weeks were significantly affected for external egg quality but did not explain the major differences, were they good or bad differences? The optimum level of isoleucine was different for each parameter. For example, For level as a factor, significantly higher egg weight was achieved at 0.69%, higher specific gravity was achieved at 0.84% isoleucine, and shell thickness was highest at 0.78% isoleucine.

**Page 12, lines 12 - 16 and page 13 lines and page 13, lines 1-9.**

**"Comparative Performance and External/Internal Egg Quality Measurements**

**The effect of level on performance parameters and external/internal egg quality measurements reported in the present study did not always agree with findings of other investigators even when the same range of isoleucine was added to layer diets. Age of layers, length of feeding, genetics, environmental factors, or imbalances of amino acid may be causative factors. As well, as noted above,** [**Liu and Selle (2017**](https://www.sciencedirect.com/science/article/pii/S0032579121000705#bib81)**) reported that the digestive dynamics of available synthetic amino acids differ from that of protein-bound ones. More work is needed to unravel the digestive dynamics because protein bound amino acids (from inexpensive sources) and available synthetic ones likely will be fed to layers more often as in the present work. Other investigators suggested that due to possible imbalances, all BCAA's should be monitored when investigating any one of them in commercial avian diets (Kidd et al., 2021). Moreover, as we reported L*W often affects many parameters. Perhaps large scale coordinated investigations across several laboratories with several types of layers over their complete laying cycle are needed to find the most appropriate level of isoleucine, valine, and leucine for layers."**

Page 7: Internal egg quality (Supplement, Table 1):

It is not clear from the table what does propositional yolk and albumen weight means? Propositional to what? Please clarify?

**See footnotes for Table 3.**

For proportional yolk weigh, the optimum level of isoleucine was 0.84%. For proportional albumen weigh, the optimum level was 0.78%. The optimum level was 0.81% for yolk index, Y: A the optimum level was 0.84%. Can they all be mentioned in the text?
Regression analysis is required to determine the optimum level.

Table3: Were these levels within the expected physiological range? Not clear. Is an increase or decrease of these significant levels a good or a bad indicator? The whole section needs to be rewritten to clarify the differences between levels? Again, which level of isoleucine resulted in desired effects?

**Authors did not mention optimum levels (requiring regression analysis) in the original or corrected work because this was not our goal. We have included reductions or increases when comparing levels as advised. It was determined that 0.72% isoleucine produced the most desired effect (for FCR).**

Table 3: A word is missing under quadratic (See the last row of the table?

**Please see corrected Table 3.**

Page 8: Discussion:
Line 11-12: Was Dong referring to egg production? Please mention.

**Correction on page 5, lines 14 - 20**.

Line 13: greater quantities? Be specific, mention what were the optimum levels for the egg production. Revise sentences.

Line 15-16: Excessive BCAAS? not clear, what is categorised as excessive levels? Revise the sentence. Which level of isoleucine can cause excessive BCAAs??

Line 16-17: Sentence needs revision: The authors need to clarify that Serotonin is an appetite suppressant. Therefore, speculation can be made that reduced feed intake at 0.75% can be associated with isoleucine level. Not clear what happened at 0.81? Please check and amend the sentence.

**Discussion has been revised.**

Page 9:
Line 3 to 4: "when less expensive feeds are used" what are less expensive feeds? Do you mean low CP feeds? Clarify the sentence.
Line 10- "Lowering isoleucine levels" What were the isoleucine levels used in Shim et al? Was the effect due to lowering of CP levels in general or was the effect specific to isoleucine levels? Not clear.

**These sentences were removed.**
Line 13-14: what were the lower levels of isoleucine used by Dong? Add the information.

**Page 11, lines 12, 13Corrections were made. These investigators used 0.54% to 0.94% isoleucine.**

**Page 14, lines 8 - 18.**

**"Total** serum albumin, digestibility, and HI titers were not affected by level. Contrary to our work, Dong et al. (2016) **reported a quadratic effect for isoleucine** levels on serum albumin. **Viral strain, susceptibility of host, age, breed, and environment (such as environmentally controlled houses versus open houses) are factors likely affecting ND titers of layers in our work and that of others (Oberlander et al., 2020). Additionally, no affect for ND titers associated with level of isoleucine in our work could be explained by presence of specific circulating antibodies produced during low levels of infections due to low endemic levels of NDV in the poultry industry worldwide (Shahid et al., 2020). As well, H9N2 virus was detected in 2015 in a Pakistani poultry worker, suggesting that H9 could be transmitted by staff to layers (Ali et al, 2019). To date, NDV and AI are prevalent in Pakistan and are included in the commercial vaccination schedule (Ditta, 2021)."**

Page 10:
Line 2-3: effect of protein sources and levels of isoleucine are two different things. Clarify the sentence.

**Corrected.**

Line 3-5: Are you comparing isoleucine with Valine? Do you expect both AA's to affect the immunoglobulins in a similar way? or is it a typo? Please check and clarify.

**Corrected. See new discussion on Page 14, lines 8 - 18.**

Line 8-10: The CP levels of this study is 16%. It may be that increase in CP level can affect ND titer. There are many other factors that can affect the ND titre? These other factors can be explored to describe your results. The sentence needs clarification.
Line 11: what are "protein deprived chicks"? Not clear.
Line 15-16: Line 14 to 17: needs revision: recommendation for further studies is very vague. Can they be specific?

**Corrected. See new discussion on Page 14, lines 8 - 18.**

Material and methods:
• Throughout the text, it should be made clear that isoleucine levels are total and not SID.
• Methods should also specify L- isoleucine quantity added in each diet to meet the required % isoleucine levels.
Page 11:
Line 6: Methods should also specify the type of cage system that was used in this study.
Line 12: the formula used to determine ileal CP digestibility should be provided in the manuscript.
Line 15: Hempe et al: year of publication is missing
Line 16: Victor and Carver, 1936: Reference is too old. Please replace it with any recent reference if possible.
Line 16: Haugh 1938: Reference is missing in the reference list. Again reference too old.
How internal and external egg parameters were analysed? The information must be added in the methods section.
**All corrected in Materials and methods with explanation or references.**

Page 12:
Line 3: Was digesta samples freeze or oven-dried. Add the information.
Statistical analysis:
• For egg production parameters it was factorial design (L*W), how can that be analysed as one way? It has to be a factorial design?
• Data must be analysed as regression analysis to confirm optimum levels of isoleucine for different parameters.

**Please see Various Forms of ANOVA. https://www.statisticssolutions.com/the-various-forms-of-anova/**

**Our goal was not to find optimum levels for each parameter.**

Reviewer #2: This is the review for the manuscript PONE-D-20-35024, entitled “Varying digestible isoleucine level to determine effects on performance, egg quality, serum biochemistry, and ileal protein digestibility in diets of young laying hens” for PLOS ONE Journal.

The present manuscript presents original data, which has been collected using appropriate standard methods. However, Minor concerns still need to be addressed or clarified.

Page 3 (Line 2): Abbreviations should be clarified when first mentioned e.g., AA = Amino acids. Clarify the three limited amino acids (including tryptophan).
Page 9 (Line 12): It is not clear why shell thickness changed with isoleucine supplementation. More explanation is required

**Page 9, lines 9 -19.**

Page 10 (Line 13): No discussions for the alteration in serum glucose content.
Page 10 (Line 19): what is 490?
Page 10 (Line 20): "and the Control + 0.69". Control or basal diet?
Page 12 (Lines 2 & 3): Many citations. Use one or two.

**Please see corrections in revised paper. Specific to references for methods, all references are critical because many analysis include several parts.**

6. PLOS authors have the option to publish the peer review history of their article ([what does this mean?](https://journals.plos.org/plosone/s/editorial-and-peer-review-process#loc-peer-review-history)). If published, this will include your full peer review and any attached files.


If you choose “no”, your identity will remain anonymous but your review may still be made public.

**No. However, I understand the policy.**
**Do you want your identity to be public for this peer review?** For information about this choice, including consent withdrawal, please see our [Privacy Policy](https://www.plos.org/privacy-policy).

Reviewer #1: No

Reviewer #2: No

[NOTE: If reviewer comments were submitted as an attachment file, they will be attached to this email and accessible via the submission site. Please log into your account, locate the manuscript record, and check for the action link "View Attachments". If this link does not appear, there are no attachment files.]

While revising your submission, please upload your figure files to the Preflight Analysis and Conversion Engine (PACE) digital diagnostic tool, <https://pacev2.apexcovantage.com/>. PACE helps ensure that figures meet PLOS requirements. To use PACE, you must first register as a user. Registration is free. Then, login and navigate to the UPLOAD tab, where you will find detailed instructions on how to use the tool. If you encounter any issues or have any questions when using PACE, please email PLOS at [figures@plos.org](mailto:figures@plos.org). Please note that Supporting Information files do not need this step.

*In compliance with data protection regulations, you may request that we remove your personal registration details at any time.* [*(Remove my information/details)*](https://www.editorialmanager.com/pone/login.asp?a=r)*. Please contact the publication office*
