# Supplementary material for: Varying isoleucine level to determine effects on performance, egg quality, serum biochemistry, and ileal protein digestibility in diets of young laying hens
Source: PLoS One. 2022 Jan 21;17(1):e0261159. doi: 10.1371/journal.pone.0261159 (PMC8782478; doi:10.1371/journal.pone.0261159)
Supplement: Attachment — Submitted filename: Reviewer comment PoneD 20 35024.docx [file pone.0261159.s001.docx]

**Reviewer’s comments on Manuscript no PONE-D-20-35024**.

The topic is very interesting and does allow knowledge transfer in the field of avian nutrition. However, the manuscript lacks certain details, which must be added. Major revision is required before it can be accepted for publication. The comments below will allow the authors to improve their manuscript:

**Abstract:**

Line 1 & 2: In control diet, 0.10 isoleucine was added to achieve 0.66% isoleucine levels. Mention the supplemental levels of isoleucine for all diets to achieve 0.66 to 0.84% isoleucine in complete feed.

Line 3 & 4: The authors must mention the control diet's crude protein value along with the ME values.

Line 6 &7: What is meant by weight change? Do you mean weight gain? Rephrase the term to clarify.

Line 12-14: Authors need to do a regression analysis to confirm the optimum levels of isoleucine for commercially important parameters. There may be different optimum levels for different parameters.

**Introduction:**

The introduction is not clear and needs to be elaborated and rewritten to justify higher isoleucine levels? What has changed, which demands higher levels?

Line 7-8: “Researchers have proposed optimum isoleucine levels to be from 550 to 660mg/hen /day”: But it is not clear how much of the supplemental isoleucine is needed in the diet to fulfil this requirement? This recommendation is for which breeds and what ages??

Line 8-9: Sentence needs revision. What do authors mean by genetic variation and age groups?

**Results:**

Table 1: was it for 20 to 30 weeks? Or 23 to 30 weeks?

Table 1: Units for Egg production and Feed intake is missing?

Table1: Change in bird weight? Not clear? I think authors means initial at minus final wt. If that is the case, then it should be mentioned as weight gain.

Table 1: Egg mass data is missing? Must add.

Page 6: line 2: Bodyweight change was significant for the week? Did it increase or decrease with age?

Page 6: Line 3: numerical differences mean nothing? The level of egg production was statistically lower for all treatments fed 0.75 to 0.84% supplemental isoleucine? Revise sentence to clarify what authors are trying to highlight?

Line 4: Highest numerical FCR? This is not true. Numerical differences should not be highlighted. Revise the sentence.

Table 2: Line 5-12: Needs revision: The authors mention that levels and weeks were significantly affected for external egg quality but did not explain the major differences, were they good or bad differences? The optimum level of isoleucine was different for each parameter. For example, For level as a factor, significantly higher egg weight was achieved at 0.69%, higher specific gravity was achieved at 0.84% isoleucine, and shell thickness was highest at 0.78% isoleucine.

Page 7: Internal egg quality (Supplement, Table 1):

It is not clear from the table what does propositional yolk and albumen weight means? Propositional to what? Please clarify?

For proportional yolk weigh, the optimum level of isoleucine was 0.84%. For proportional albumen weigh, the optimum level was 0.78%. The optimum level was 0.81% for yolk index, Y: A the optimum level was 0.84%. Can they all be mentioned in the text?

Regression analysis is required to determine the optimum level.

Table3: Were these levels within the expected physiological range? Not clear. Is an increase or decrease of these significant levels a good or a bad indicator? The whole section needs to be rewritten to clarify the differences between levels? Again, which level of isoleucine resulted in desired effects?

Table 3: A word is missing under quadratic (See the last row of the table?

**Page 8: Discussion:**

Line 11-12: Was Dong referring to egg production? Please mention.

Line 13: greater quantities? Be specific, mention what were the optimum levels for the egg production. Revise sentences.

Line 15-16: Excessive BCAAS? not clear, what is categorised as excessive levels? Revise the sentence. Which level of isoleucine can cause excessive BCAAs??

Line 16-17: Sentence needs revision: The authors need to clarify that **Serotonin** is an **appetite** suppressant. Therefore, speculation can be made that reduced feed intake at 0.75% can be associated with isoleucine level. Not clear what happened at 0.81? Please check and amend the sentence.

Page 9:

Line 3 to 4: "when less expensive feeds are used" what are less expensive feeds? Do you mean low CP feeds? Clarify the sentence.

Line 10- "Lowering isoleucine levels" What were the isoleucine levels used in Shim et al? Was the effect due to lowering of CP levels in general or was the effect specific to isoleucine levels? Not clear.

Line 13-14: what were the lower levels of isoleucine used by Dong? Add the information.

Page 10:

Line 2-3: effect of protein sources and levels of isoleucine are two different things. Clarify the sentence.

Line 3-5: Are you comparing isoleucine with Valine? Do you expect both AA's to affect the immunoglobulins in a similar way? or is it a typo? Please check and clarify.

Line 8-10: The CP levels of this study is 16%. It may be that increase in CP level can affect ND titer. There are many other factors that can affect the ND titre? These other factors can be explored to describe your results. The sentence needs clarification.

Line 11: what are "protein deprived chicks"? Not clear.

Line 15-16: Line 14 to 17: needs revision: recommendation for further studies is very vague. Can they be specific?

**Material and methods:**

- Throughout the text, it should be made clear that isoleucine levels are total and not SID.
- Methods should also specify L- isoleucine quantity added in each diet to meet the required % isoleucine levels.

Page 11:

Line 6: Methods should also specify the type of cage system that was used in this study.

Line 12: the formula used to determine ileal CP digestibility should be provided in the manuscript.

Line 15: Hempe et al: year of publication is missing

Line 16: Victor and Carver, 1936: Reference is too old. Please replace it with any recent reference if possible.

Line 16: Haugh 1938: Reference is missing in the reference list. Again reference too old.

How internal and external egg parameters were analysed? The information must be added in the methods section.

Page 12:

Line 3: Was digesta samples freeze or oven-dried. Add the information.

**Statistical analysis:**

- For egg production parameters it was factorial design (L*W), how can that be analysed as one way? It has to be a factorial design?
- Data must be analysed as regression analysis to confirm optimum levels of isoleucine for different parameters.
